# Investigation of the Phenolic Component Bioavailability Using the In Vitro Digestion/Caco-2 Cell Model, as well as the Antioxidant Activity in Chinese Red Wine

**DOI:** 10.3390/foods11193108

**Published:** 2022-10-06

**Authors:** Chunming Xu, Lingqiang Kong, Yuan Tian

**Affiliations:** 1 School of Light Industry, Beijing Technology and Business University, Beijing 100048, China; 2 Key Laboratory of Cleaner Production and Integrated Resource Utilization of China National Light Industry, Beijing Technology and Business University, Beijing 100048, China

**Keywords:** red wine, PCs, bioavailability, in vitro digestion, structural equation

## Abstract

Red wine is a well-known alcoholic beverage, and is known to have phenolic compounds (PCs), which contribute to its antioxidant activity and have other beneficial advantages for human health. The aim of this study was to evaluate the effect of the simulated gastro-intestinal digestion and the Caco-2 transepithelial transport assay on the PCs, bioavailability, and the antioxidant capacity of red wines. The contents of PCs in red wine were significantly reduced during most of the digestion phases. Phenolic acid had the greatest permeability, while the flavonols had the weakest. The bioavailability of PCs ranged from 2.08 to 24.01%. The result of the partial least squares structural equation model showed that the three phenols were positively correlated with the antioxidant activity of red wine. The contribution of anthocyanins was the largest (0.8667).

## 1. Introduction

In recent years, red wine has received more and more attention as a popular alcoholic beverage worldwide [1,2]. Its consumption increases gradually with the development of a nation’s economy [3]. The total phenolic content of red wine is estimated to be between 2 and 6 mg/mL [4,5]. These PCs exhibit a wide range of activities, including antioxidant, antihypertensive, anti-inflammatory, anti-microbial, cardioprotective, and neuroprotective properties [6,7,8,9,10].

The bioaccessibility and bioavailability of PCs determine their potential value as bioactive agents. An in vitro digestion model was recently developed to assess the bioaccessibility of target molecules in a variety of products [11]. Bioavailability refers to the proportion of distinct digestive samples that carry target compounds from the intestinal cavity into the bloodstream in the human body [12]. Although in vivo research can produce more advanced information and apply it to human health, for ethical and cost reasons, great effort has been made to search alternative methods for plant secondary metabolite development. The method of using animals to evaluate the metabolism and/or toxicity of plant secondary metabolites has been carefully considered in research and related applications. Animal studies have been conducted mainly to determine the compound pharmacokinetics and toxicological data. At present, the in vitro assays based on cell cultures are some of the most important among all of the in vitro assays for performing in vitro–in vivo extrapolation. There are many examples on cell-based data that are incorporated into mathematical modeling in order to predict the in vivo result accurately and successfully [13]. The Caco-2 cell line has long been used to investigate nutrient bioavailability in various food matrices [14]. It is a sophisticated model to study the cross-transepithelial transport of substances in the human intestine and the mechanics of the intestinal barrier. Therefore, it was used in this study to investigate bioavailability.

Under normal metabolic conditions, the human body produces reactive oxygen species (ROS) [15]. Extensive defense mechanisms are present to counteract the effects of these oxidizing agents [16]. Large amounts of ROS in the body can cause oxidative stress, affect biological components, cause genetic damage and mutations, and induce cell death [17]. Effective alternatives to conventionally-used antioxidant enzymes must be identified to reduce ROS in the body. Previous studies on the antioxidant activity of PCs in red wine mainly considered the overall antioxidant activity of the sample solution after in vitro digestion [18,19]. However, PCs must be absorbed by small intestinal cells before they can exert an antioxidant activity in vivo; the ability of different kinds of PCs to penetrate these cells differs [20]. The antioxidant activity of the sample solution after in vitro simulation digestion may not reflect the actual antioxidant activity, and is a reflection of the total polyphenols. However, different kinds of PCs do not have the same antioxidant activity. In this study, we tried to clarify the changes in the PCs in red wine before and after simulated gastrointestinal digestion, along with the Caco-2 transepithelial transport assay after simulated digestion. The bioavailability of different PCs was explored. The partial least square structural equation model was used to reveal the relationship between different families of PCs and their antioxidant activities for the first time.

## 2. Materials and Methods

### 2.1. Chemicals

Standards of (+)-catechin, malvidin-3-glucoside, caffeic acid, quercetin, iso-quercetin, and gallic acid were purchased from Sigma Chemical Co. (St. Louis, MO, USA). The purity grade of all standards was greater than 99%.

Porcine amylase, porcine pepsin, porcine pancreatin, and fetal bovine serum were purchased from Sigma Chemical Co. (St. Louis, MO, USA).

Formic acid (HPLC-grade), acetonitrile (HPLC-grade), ethanol, Dulbecco’s Modified Eagle Medium (DMEM)culture medium, methyl thiazolyl tetrazolium, EDTA, penicillin, streptomycin, DPPH, acetic acid, TPTZ, FeCl_3_, ABTS, and potassium persulfate were purchased from a commercial reagent company (Omic Inc., Beijing, China). In addition to HPLC-grade reagents, all of the other reagents were of analytical grade. Ultrapure water was obtained from a Milli-Q water purification system (Millipore Corp., Bedford, MA, USA).

### 2.2. Wine Samples

Three red wines were obtained from three central grape-planting regions in China, namely Yantai, Helan Mountain, and Tianshan Mountain. The grape cultivar was *Cabernet Gernischt*. The three brands of red wine corresponded to the three grape-planting regions: Cabernet, Helanshan, and Tianzhu. The wine from Cabernet comprised 12% alcohol, pH 3.6, and free SO_2_ of 45 ppm. The wine from Helanshan was composed of 13% alcohol, with a pH of 3.5 and a free SO_2_ amount of 45 ppm. The wine from Tianzhu contained 13% alcohol, with a pH of 3.4 and a free SO_2_ amount of 40 ppm. The age of the wine used was three years. The wine bottles were stored upside down at a specific angle in the dark at a low temperature, and the appropriate dilution was determined immediately after opening.

### 2.3. In Vitro Simulation Digestion Procedure

The digestion procedure was carried out by adapting the method of Brodkorb et al. and Gómez et al. [21,22]. First, 10 mL of a 0.85% (*w*/*v*) NaCl solution was mixed with 15 mL of a sample of red wine and was preheated for 15 min at 37 °C. One milliliter (50 units/mL) of porcine amylase was prepared in a 15 mM Na₃PO₄ buffer solution (pH adjusted to 6.8) containing 1.5 mM CaCl_2_, and it was added to the red wine solution. After 5 min, 4.5 mL of 0.12 M HCl was added to the mixture to adjust the pH to 2.4. One milliliter of porcine pepsin (0.02 mg/L) was dissolved in a 20 mM HCl solution. The mixture was then maintained at 37 °C for 120 min. The digestion mixture was then centrifuged at 8000× *g* for 15 min to obtain samples from the gastric digestion phase. The gastric samples were then reacted with a mixture containing 5 mL each of bile salt (0.15 mg/L) and porcine pancreatin (20 mg/L). The mixture was incubated at pH 6.5 at 37 °C for 3 h before being centrifuged. The extracts of the intestinal phase were the supernatants. The same operation was done three times, and each sample was stored in a refrigerator (−20 °C) immediately for further analysis.

### 2.4. Cell Culture

Caco-2 cells were inoculated following the method of Hubatsch et al. [23]. Briefly, a 5% *v*/*v* of CO_2_ incubator at 37 °C was used to house the cells. DMEM culture medium (pH = 7.4) supplemented with 9% *v*/*v* fetal bovine serum, 1% *v*/*v* antibiotic mixture (penicillin and streptomycin), and 1.2% *v*/*v* non-essential amino acids was used. Caco-2 cells were sub-cultured when they reached 80–90% of the adherent growth by treating them with a 0.05% *v*/*v* of trypsin–EDTA solution. The culture between passages 35 and 40 was used for the following experiment.

### 2.5. Transport Experiments Using the Caco-2 Cell Model

Transport experiments were performed according to the method of Wu et al. with slight modifications [24]. First, 1.0 × 10^5^ Caco-2 cells were inoculated onto 12 mm transparent cell culture plates (Transwell). Media were carefully injected into the apical (0.5 mL) and basolateral (1.5 mL) sides of the Transwell plates. The cell culture medium had to be replaced every other day until the cells formed a monolayer. The integrity and transport ability of the Caco-2 cell monolayer were evaluated by measuring the trans-epithelial electrical resistance (TEER) with a MilliCell voltammeter (Millicell ERS-2, Merck Millipore, Billerica, MA, USA). The TEER value exceeded 100 from day 14 to 21 after inoculation, indicating that the cell integrity in the monolayer met the requirements for the subsequent experiments. The monolayer formation was confirmed by the fact that the calculated TEER was higher than 400/cm^2^. The methyl thiazolyl tetrazolium (MTT) cell viability test revealed that the phenols did not affect the viability of intestinal cells. When the cell activity exceeded 0.9, a transport experiment was conducted. The apical side was injected with a digested red wine sample (0.5 mL) and was thoroughly mixed with anHank’s Balanced Salt Solution (HBSS) buffer solution. Only 1.5 mL of the buffer solution was added to the basolateral side of the monolayer. The cells were then cultured in a 37 °C, 5% CO_2_ incubator. Then, 0.25 mL samples were separately taken from the basolateral side of the monolayer. An equal volume of HBSS buffer was then added every half hour from the beginning of the culture until the end of the culture at 6 h. The collected samples were subsequently filtered and placed in the sample bottle for further HPLC analysis. This experiment was repeated several times to collect enough samples for the next antioxidant test. The apparent permeability coefficient (P_app_) is typically used to characterize the transport capacity of substances and was calculated using the following equation:

(1) $$P_{\mathrm{app}}\left(\mathrm{cm}/s\right)=\mathrm{dC}/\mathrm{dt}\times V/A\cdot C_{0}$$

dC/dt represents the concentration change value per unit time for PCs on the basolateral side (mM/s), V represents the volume of the basolateral side (mL), A represents the membrane surface area (cm^2^), and C_0_ represents the initial PCs concentration on the apical side (mM).

### 2.6. Determination of the Phenolic Profile

The Pati method was adopted, with slight modifications, to measure the content of the PCs [25]. The Agilent 1100 series HPLC instrument was used; it was equipped with a degasser, quaternary pump solvent delivery, a DAD system, and a single quadrupole mass detector coupled with an electrospray ionization LC–MS interface. Briefly, th samples were injected into a Zorbax SB C18 reversed-phase column (5 µm,150 × 2.1 mm, Agilent Technologies, Santa Clara, CA, USA) using an autosampler after filtering through 0.45 µm cellulose acetate syringe filters. The column temperature was kept at 30 °C, the injection volume was 10 µL, and the flow rate was 0.3 mL/min. The eluents were water and formic acid in a 99:1 (*v*/*v*) (A) ratio and 100% acetonitrile (B). The gradient elution procedure was as follows: (1) 3% B solvent at 0 min, (2) 3 to 16% B solvent at 10 min, (3) 16 to 20% B solvent at 30 min, and (4) 20% B solvent maintained until the procedure was completed. The MS configuration was as follows: ESI, negative ion mode, nebulizer pressure of 30 psi, a dry gas flow rate of 10 mL/min, dry gas temperature of 350 °C, and a scan rate of 100~1200 m/z. PCs were determined according to their retention times, UV/VIS spectra, and high-resolution MS spectra, in addition to a comparison with the standards. Quantification was carried out using the commercial standards of (+)-catechin, malvidin-3-glucoside, caffeic acid, quercetin, iso-quercetin, and gallic acid in a range from 0.015 mg/L to 300 mg/L. If a standard was unavailable, a structurally related compound was used. Putative annotations were made using spectral features and literature information on the chromatographic properties and mass spectra records from the metabolome database. A standard curve was drawn using methanol solutions with different concentrations as the standards. The limits of detection and quantitation were calculated according to the signal-to-noise ratio (limit of detection: signal-to-noise ratio ≥ 3; limit of quantitation: signal-to-noise ratio ≥ 10). The limit of quantitation ranged from 0.015 to 0.085 mg/L. The CV was below 11%. PCs All of the samples were determined in triplicate.

### 2.7. Assessment of In Vitro Antioxidant Potential

#### 2.7.1. DPPH Radical-Scavenging Activity

The red wine sample was measured using the method of Sabeena with slight modifications, for the determination of the DPPH radical scavenging activity [26]. The solution was prepared as follows: 7.8864 mg of analytically pure DPPH was dissolved in 75% ethanol to obtain a total volume of 100 mL. Three milliliters of each red wine sample were homogeneously blended with equal amounts of DPPH solution, and the mixture was kept at 37 °C for half an hour in the dark. Absorbance was recorded at 517 nm.

DPPH radical scavenging activity (%) = (A_control_ − A_sample_)/A_control_ × 100 (2)

“A_sample_” is the absorbance of the sample in the DPPH solution and “A_control_” is the absorbance of the DPPH solution in water.

#### 2.7.2. Ferric Reducing Antioxidant Power (FRAP)

The ferric-reducing activities of the red wines sample were assessed as follows [27]. The FRAP solution was prepared as follows: 0.3 M acetic acid buffer (pH 3.6), 10 mM TPTZ solution, and 20 mM FeCl_3_ solution were mixed at a ratio of 10:1:1 and used immediately after preparation. The red wine sample solution (3 mL) was homogeneously mixed with 3 mL of fresh FRAP reagent and was incubated in the dark at 37 °C at a constant temperature for 10 min. Absorbance was recorded at 593 nm.

FRAP radical scavenging activity (%) = (A_control_ − A _sample_)/A_control_ × 100 (3)

“A_sample_” is the absorbance of the sample in the FRAP solution and “A_control_” is the absorbance of water in the FRAP solution.

#### 2.7.3. ABTS Free Radical Scavenging Activity

To investigate the ABTS radical scavenging activity of the red wine, the ABTS assay was performed using the method of Abdel-Hamid [28]. The ABTS solution was prepared as follows: 38.4 mg ABTS was dissolved in water, to obtain a volume of 10 mL. Potassium persulfate (13.4 mg) was dissolved in water to obtain a final volume of 10 mL. The two solutions were mixed in a ratio of 1:1 and were diluted 20 times with PBS (pH = 7.4) before use. The red wine sample (3 mL) was mixed with 3 mL of ABTS solution. After incubating for half an hour under dark conditions, absorbance was measured at 405 nm.

ABTS radical scavenging activity (%) = (A_control_ − A_sample_)/A_control_ × 100 (4)

“A_sample_” is the absorbance of the sample in the ABTS solution and “A_control_” is the absorbance of water in the ABTS solution.

### 2.8. Statistical Analysis

All of the experiments and analyses were repeated three times to obtain the average. The final results were presented in the form of the mean ± standard deviation. The experimental groups were compared by one-way ANOVA, and the mean difference was calculated by Tukey’s multiple comparison test (*p* < 0.05). The relationships between the antioxidant activity and the different kinds of polyphenols were estimated using a partial least square structural equation model.

## 3. Results and Discussion

### 3.1. Change in PCs during the Simulated Gastrointestinal Digestion and Transmembrane Process

PCs are well-known for their antioxidant potential and are abundant in fruits and vegetables. During the in vitro simulation gastrointestinal digestion phase, to observe the changes in individual PCs, 23 compounds were investigated using HPLC; these compounds included eight anthocyanins, three flavanols, seven flavonols, and five phenolic acids. Table 1 depicts the change in the content of PCs in various red wine samples before and after simulation digestion. The simulated digestion process significantly reduced most phenolic substances. Among the anthocyanins and flavanols, only delphinidin-3-glucoside and epicatechin increased by 20.6 and 14.5% after digestion in Tianzhu; they increased by 28.3 and 14.7% after digestion in Helanshan. In the flavonols category, only the iso-quercetin content in Tianzhu was slightly increased by 9.8% after digestion. In contrast, in the phenolic acids category, the fertaric acid content in the three red wines increased by 41.2, 22.3, and 127.0% after digestion, respectively, which could be due to the action of different enzymes or bile salts on the red wine matrix, resulting in the release in bound PCs [29]. These results were consistent with those of Zhang (2017) [30] and Chiat (2020) [31].

During the simulation of gastric digestion, polyphenols were exposed to acidic gastric juice and pepsin, which accelerated the hydrolysis of polymerized polyphenols and converted them to monomers or aglycones [32]. The bonds between the PCs and proteins, fibers, or sugar residues were broken, causing changes in the molecular weight (MW), water solubility, and spatial structure, thereby reducing the total content of PCs upon gastric digestion [33]. The change in PCs during the intestinal digestion phase may be due to their rapid degradation in an alkaline environment, as well as their possible interaction with bile acids or other compounds [34]. These interactions between PCs and dietary constituents can result in covalent compounds that may reduce or increase the bioaccessibility of PCs [35]. Red wine contains many polysaccharides, such as soluble fibers, that are often bonded with PCs [36]. The pepsin and gastric acid environments have no significant effect on the degradation of flavonols and phenolic acids [37]. Malvidin-3-glycoside, quercetin-3-glycoside, and ethyl gallate constituted the highest content of PCs in red wine. The latter two compounds are known to be more stable under acidic conditions [37]. The molecular structure of PCs generally comprises acidic phenolic hydroxyl groups, which are unstable under alkaline conditions. Therefore, quick transformation in chemical bonds may take place to produce new compounds with various biological activities and bioavailabilities [37]. During the in vitro gastrointestinal digestion step, complex biotransformation processes such as glucuronidation, methylation, and sulfation occur, promoting the formation of several chemical compounds, such as phenolic acids. Many factors can influence the loss of anthocyanins and flavanols during intestinal digestion. Firstly, the pH value improves polyphenol degradation in the small intestine [38]. Secondly, the interaction of polyphenols with other components may hinder absorption. Thirdly, the oxidation and polymerization of phenols in the small intestine produce new phenolic by-products. Lastly, the way enzymes work changes the structure of molecules, which may render polyphenols less soluble, making it challenging for the body to absorb them [34]. Therefore, the interaction between phenolic substances and digestive enzymes, as well as changes in pH values, may cause a shift in the concentration of phenolic substances during gastrointestinal digestion. A previous study on sweet oranges (*Citrus sinensis*) revealed a similar phenomenon during gastrointestinal digestion [39].

### 3.2. Caco-2 Transepithelial Transport Assay

In the last decade, the Caco-2 cell monolayer model has usually been used to evaluate the intestinal transport of PCs [40,41,42]. As shown in Table 1, the transport of each of the PCs from the apical side of the Caco-2 monolayer to its basolateral side was evaluated. The apparent permeability coefficients of PCs are shown in Table 2; there were no marked differences among the apparent permeability coefficients of the different red wine samples. Phenolics acid had the greatest permeability, and flavonols had the weakest permeability. The permeabilities of anthocyanins and flavanols were between those of the phenolic acids and flavanols. Gallic acid had the greatest permeability (32.8 ± 4.13 cm/s) among all of the PCs. Rastogi and Jana (2016) [43] similarly reported a higher permeability for caffeic and gallic acids in the Caco-2 monolayers model. Because of their distinct molecular structures, different PCs have different affinities for membrane and transcellular transporters upon infiltration into the Caco-2 cells. Flavonols may have a low permeability because of their low affinity for glucose transporters and slow passive diffusion.

**Figure 1:**
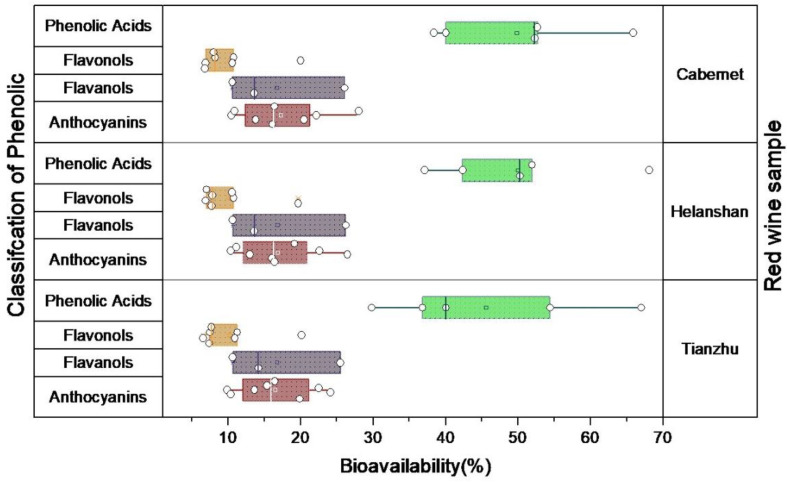
Bioavailability of four kinds of PCs.

In addition to their transmembrane ability, the structural integrity of PCs influences their bioavailability. Bioavailability is defined as the fraction of a chemical that is available for biological action. In this paper, bioavailability was calculated from the ratio of the transmembrane content of phenolic acids and their undigested form. The bioavailability of four types of PCs is depicted in Figure 1. The bioavailability of PCs in the three red wine samples ranged from 2.08 to 24.01%. The flavonol bioavailability was the lowest, whereas the anthocyanins and flavanols had a comparable bioavailability [44]. The bioavailability of gallic acid in the Cabernet red wine sample was the highest among the four types of PCs, with a maximum bioavailability of 24.01%. In the cell culture, the stability of PCs was significantly lower than in the organic solvents, indicating that they were easily degraded, resulting in a very low bioavailability. Because of the poor lipid solubility, the transmembrane transport of PCs was slow. A cell membrane protein affected the bioavailability of PCs [45]. In addition, previous research has shown that the bioavailability of anthocyanins is low, while phenolic acids, such as ethyl gallate and other small molecular weight phenolic substances, are more easily absorbed [46,47,48]. These findings of our study are consistent with those of previous studies.

### 3.3. Antioxidant Activity

#### 3.3.1. DPPH Radical-Scavenging Activity

Oxidative stress is defined as a state when the balance between the cellular antioxidant defense and oxidants is destroyed. The functional activity of PCs varied with the composition of samples that had been digested. As shown in Table 3, there were significant differences in DPPH scavenging activity among the red wines. This decreased significantly during the digestion and transmembrane processes. During the undigested phase, all samples for the Chinese red wine had the highest DPPH-scavenging activity. The peak value of 82.7 ± 4.21% occurred during the undigested phase of the Cabernet samples. Our research confirmed the findings of a previous study on raspberries [49], which similarly reported that phenolics reflected the overall antioxidant activity of digestion for the red wine samples.

#### 3.3.2. FRAP Radical-Scavenging Activity

Table 3 shows the FRAP radical scavenging activity during the different phases of the digestion of red wine. Simulation digestion decreased the FRAP radical scavenging activity significantly. After digestion, the Tianzhu, Helanshan, and Cabernet wines presented decreased FRAP radical scavenging activity by 5.9, 11.0, and 9.6%, respectively. Furthermore, Cabernet showed the highest FRAP radical scavenging activity in the undigested phase. The FRAP radical scavenging activity in the three red wine samples differed significantly before and after the transmembrane process.

When antioxidants oxidize ABTS, they produce colored products. These products are ABTS cationic free radicals that can be measured using spectrophotometry. Antioxidants may influence the onset of a reaction and are positively correlated with antioxidant activity. As shown in Table 3, the trend of the ABTS radical scavenging activity during simulation digestion was consistent with the antioxidant activity of DPPH and FRAP. The transition from the undigested to the transmembrane phase decreased the ABTS activity in all of the samples by approximately seven-fold. A similar radical scavenging behavior has been observed in plums [50].

#### 3.3.4. Correlation between PCs and Antioxidant Activity

**Figure 2:**
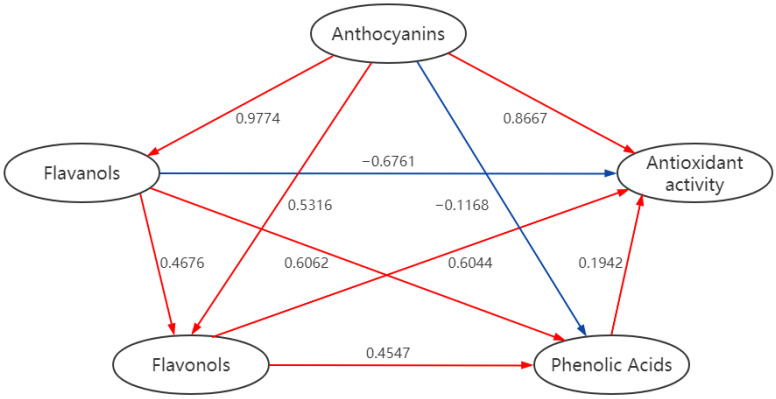
Partial least square structural equation mode.

The correlation between PCs and antioxidant activity in red wine was clarified by the partial least square structural equation model. As shown in Figure 2, three families of PCs, including anthocyanins, phenolic acids, and flavonols, were positively correlated with the antioxidant activity of red wine. However, the content of flavanols showed a negative correlation with the antioxidant activity. Correlations existed among the four phenolic components; anthocyanins and flavonols had the strongest correlation with a coefficient of 0.9774. In contrast, anthocyanins and phenolic acids were weakly correlated, with a correlated coefficient of −0.1168. According to the partial least square structural equation model, changes in the four phenolic components in the simulated digestion stage were correlated. At the same time, the contribution of the four families of PCs to the final antioxidant activity also differed, and the contribution of anthocyanins was the largest. Changes in the ROS levels or plasma antioxidant levels can be used to characterize the state of antioxidation, which is related to anthocyanins. The various substituents on the tricyclic structure of anthocyanins can exert a direct antioxidant activity. Hydroxyl (−OH) groups can provide hydrogen donors for redox reactions, while methoxy (−OCH_3_) groups may play an important role as intramolecular electron donors [51]. When lipid peroxy radicals are present, anthocyanins transform into free radical intermediates that may inhibit the peroxidation process [52]. Therefore, future studies on the antioxidant activity should focus on the changes in the anthocyanin content.

## 4. Conclusions

Simulated gastrointestinal digestion significantly affected the stability of PCs in the three red wines. It can be seen from the Caco-2 transepithelial transport assay after simulated digestion that PCs with different molecular structures had different effects on the permeabilities and bioavailability. Three antioxidant activity evaluation methods showed that the trends of change in the antioxidant activity in the three red wines were consistent in the different treatment phases. The partial least squares structural equation model was introduced for the first time to evaluate the contribution of different phenols regarding the antioxidant activity of red wine, and relatively ideal results were obtained.

## Figures and Tables

**Table 1:** PCs content in the red wines at different phases.

PC Contents (mg/L)		Tianzhu			Helanshan			Cabernet		
		Undigested	After Digestion	Transmembrane	Undigested	After Digestion	Transmembrane	Undigested	After Digestion	Transmembrane
Delphinidin-3-glc	Anthocyanins	2.38 ± 0.12 ^b^	2.87 ± 0.11 ^c^	0.57 ± 0.02 ^a^	2.05 ± 0.09 ^b^	2.63 ± 0.10 ^c^	0.53 ± 0.02 ^a^	3.45 ± 0.15 ^c^	2.96 ± 0.09 ^b^	0.59 ± 0.02 ^a^
Petunidin-3-glc	Anthocyanins	6.21 ± 0.29 ^c^	5.33 ± 0.21 ^b^	1.06 ± 0.04 ^a^	5.86 ± 0.27 ^c^	4.98 ± 0.19 ^b^	1.40 ± 0.05 ^a^	8.29 ± 0.31 ^c^	5.79 ± 0.27 ^b^	1.56 ± 0.07 ^a^
Peonidin-3-glc	Anthocyanins	4.92 ± 0.22 ^b^	4.31 ± 0.19 ^b^	0.86 ± 0.03 ^a^	4.66 ± 0.21 ^c^	4.02 ± 0.18 ^b^	1.40 ± 0.06 ^a^	5.37 ± 0.27 ^b^	5.11 ± 0.24 ^b^	1.72 ± 0.07 ^a^
Malvidin-3-glc	Anthocyanins	163.2 ± 6.04 ^c^	103.8 ± 5.71 ^b^	20.68 ± 1.05 ^a^	113.2 ± 4.19 ^c^	88.2 ± 3.97 ^b^	35.55 ± 1.46 ^a^	208.32 ± 10.83 ^c^	153.43 ± 5.83 ^b^	62.38 ± 3.24 ^a^
Delphinidin-3-acglc	Anthocyanins	0.48 ± 0.016 ^c^	0.22 ± 0.008 ^b^	0.04 ± 0.002 ^a^	0.33 ± 0.013 ^c^	0.14 ± 0.007 ^b^	0.02 ± 0.001 ^a^	0.67 ± 0.03 ^c^	0.29 ± 0.014 ^b^	0.04 ± 0.002 ^a^
Petunidin-3-acglc	Anthocyanins	1.42 ± 0.05 ^c^	1.17 ± 0.04 ^b^	0.23 ± 0.008 ^a^	1.37 ± 0.05 ^c^	0.98 ± 0.05 ^b^	0.18 ± 0.005 ^a^	1.87 ± 0.08 ^c^	1.05 ± 0.05 ^b^	0.19 ± 0.008 ^a^
Malvidin-3-acglc	Anthocyanins	51.6 ± 2.48 ^c^	39.3 ± 1.30 ^b^	7.83 ± 0.23 ^a^	45.5 ± 2.09 ^c^	30.5 ± 0.92 ^b^	6.06 ± 0.25 ^a^	63.4 ± 3.36 ^c^	39.8 ± 1.51 ^b^	8.02 ± 0.38 ^a^
Peonidin-3-acglc	Anthocyanins	5.32 ± 0.29 ^c^	3.88 ± 0.16 ^b^	0.77 ± 0.03 ^a^	4.56 ± 0.23 ^c^	3.21 ± 0.14 ^b^	0.44 ± 0.02 ^a^	6.87 ± 0.27 ^c^	3.46 ± 0.15 ^b^	0.47 ± 0.02 ^a^
Catechin	Flavanols	35.2 ± 1.80 ^c^	16.7 ± 0.75 ^b^	3.33 ± 0.15 ^a^	30.42 ± 1.58 ^c^	15.42 ± 0.83 ^b^	1.90 ± 0.07 ^a^	47.6 ± 1.62 ^c^	25.4 ± 0.86 ^b^	3.16 ± 0.15 ^a^
Epicatechin	Flavanols	18.6 ± 0.95 ^b^	21.3 ± 0.98 ^c^	4.24 ± 0.20 ^a^	16.33 ± 0.60 ^b^	18.17 ± 0.81 ^b^	3.35 ± 0.16 ^a^	22.1 ± 0.86 ^c^	10.2 ± 0.38 ^b^	1.86 ± 0.10 ^a^
Procyanidin dimer	Flavanols	8.31 ± 0.26 ^b^	7.64 ± 0.32 ^b^	1.52 ± 0.08 ^a^	7.62 ± 0.24 ^c^	5.98 ± 0.27 ^b^	0.62 ± 0.02 ^a^	9.67 ± 0.34 ^c^	5.64 ± 0.26 ^b^	0.58 ± 0.03 ^a^
Myricetin-3-glc	Flavonols	51.42 ± 1.59 ^b^	43.22 ± 1.34 ^b^	8.61 ± 0.43 ^a^	39.43 ± 1.93 ^c^	28.45 ± 1.51 ^b^	0.64 ± 0.02 ^a^	60.54 ± 2.91 ^c^	36.91 ± 1.10 ^b^	0.81 ± 0.04 ^a^
Astilbin	Flavonols	38.11 ± 1.87 ^c^	25.36 ± 1.32 ^b^	5.05 ± 0.19 ^a^	30.32 ± 1.46 ^b^	24.63 ± 1.13 ^b^	0.93 ± 0.04 ^a^	40.12 ± 1.69 ^c^	28.22 ± 1.07 ^b^	1.11 ± 0.05 ^a^
Laricitrin-3-glc	Flavonols	36.54 ± 1.10 ^c^	28.63 ± 0.86 ^b^	5.70 ± 0.29 ^a^	28.34 ± 1.02 ^c^	18.76 ± 0.81 ^b^	0.55 ± 0.03 ^a^	45.33 ± 1.81 ^c^	29.78 ± 1.28 ^b^	0.84 ± 0.04 ^a^
Quercetin-3-glc	Flavonols	195.65 ± 8.41 ^b^	188.45 ± 8.29 ^b^	37.54 ± 1.95 ^a^	167.35 ± 7.20 ^b^	145.43 ± 5.38 ^b^	5.10 ± 0.28 ^a^	256.17 ± 10.76 ^b^	202.22 ± 10.52 ^b^	7.47 ± 0.23 ^a^
Isoquercetin	Flavonols	17.43 ± 0.85 ^b^	19.13 ± 0.69 ^b^	3.81 ± 0.18 ^a^	14.23 ± 0.57 ^b^	12.01 ± 0.37 ^b^	0.72 ± 0.04 ^a^	21.45 ± 1.12 ^c^	13.46 ± 0.59 ^b^	0.83 ± 0.04 ^a^
Syringetin-3-glc	Flavonols	34.75 ± 1.11 ^b^	30.19 ± 1.03 ^b^	6.01 ± 0.22 ^a^	28.46 ± 1.28 ^c^	20.17 ± 1.01 ^b^	0.91 ± 0.03 ^a^	46.97 ± 2.58 ^c^	26.34 ± 0.90 ^b^	1.23 ± 0.04 ^a^
Isorhamnetin-3-glc	Flavonols	15.34 ± 0.80 ^c^	9.88 ± 0.35 ^b^	1.97 ± 0.07 ^a^	11.43 ± 0.55 ^b^	9.87 ± 0.47 ^b^	0.69 ± 0.03 ^a^	19.42 ± 0.95 ^c^	10.82 ± 0.32 ^b^	0.74 ± 0.03 ^a^
Gallic acid	Phenolic Acids	9.36 ± 0.37 ^c^	6.91 ± 0.26 ^b^	1.38 ± 0.05 ^a^	8.25 ± 0.25 ^c^	5.11 ± 0.25 ^b^	2.53 ± 0.14 ^a^	10.99 ± 0.52 ^b^	9.08 ± 0.44 ^b^	4.61 ± 0.23 ^a^
Fertaric acid	Phenolic Acids	2.91 ± 0.14 ^b^	4.11 ± 0.18 ^c^	0.82 ± 0.03 ^a^	1.48 ± 0.07 ^b^	1.81 ± 0.10 ^c^	0.81 ± 0.04 ^a^	1.37 ± 0.05 ^a^	3.11 ± 0.12 ^b^	1.40 ± 0.07 ^a^
Ethyl gallate	Phenolic Acids	70.98 ± 2.20 ^c^	40.83 ± 1.51 ^b^	8.13 ± 0.28 ^a^	61.38 ± 2.09 ^c^	30.18 ± 0.94 ^b^	8.64 ± 0.36 ^a^	60.22 ± 2.65 ^c^	38.42 ± 1.88 ^b^	10.70 ± 0.33 ^a^
Caftaric acid	Phenolic Acids	6.45 ± 0.34 ^c^	5.35 ± 0.23 ^b^	1.07 ± 0.03 ^a^	5.84 ± 0.26 ^c^	3.46 ± 0.19 ^b^	0.49 ± 0.02 ^a^	4.77 ± 0.20 ^c^	3.01 ± 0.17 ^b^	0.43 ± 0.01 ^a^
Coutaric acid	Phenolic Acids	4.32 ± 0.19 ^c^	3.21 ± 0.15 ^b^	0.64 ± 0.03 ^a^	3.84 ± 0.18 ^c^	2.97 ± 0.15 ^b^	0.61 ± 0.03 ^a^	2.85 ± 0.11 ^c^	1.96 ± 0.08 ^b^	0.38 ± 0.02 ^a^

Three replicates were used for the experiment. Means with different letters show the remarkable differences according to the ANOVA (*p* < 0.05).

**Table 2:** Apparent permeability coefficients of PCs.

PCs		Apparent Permeability Coefficients (×10^−6^ cm/s)		
		Tianzhu	Helanshan	Cabernet
Delphinidin-3-glc	Anthocyanins	12.45 ± 2.11 ^a^	12.59 ± 2.64 ^a^	12.38 ± 2.37 ^a^
Petunidin-3-glc	Anthocyanins	17.13 ± 3.01 ^a^	17.56 ± 3.32 ^a^	16.89 ± 2.96 ^a^
Peonidin-3-glc	Anthocyanins	21.42 ± 3.89 ^a^	21.69 ± 3.52 ^a^	21.13 ± 3.63 ^a^
Malvidin-3-glc	Anthocyanins	25.37 ± 4.11 ^a^	25.19 ± 3.99 ^a^	25.41 ± 47.26 ^a^
Delphinidin-3-acglc	Anthocyanins	8.78 ± 1.44 ^a^	8.64 ± 1.38 ^a^	8.57 ± 1.26 ^a^
Petunidin-3-acglc	Anthocyanins	11.46 ± 2.05 ^a^	11.78 ± 2.11 ^a^	11.53 ± 2.01 ^a^
Malvidin-3-acglc	Anthocyanins	12.37 ± 2.45 ^a^	12.42 ± 2.23 ^a^	12.59 ± 2.16 ^a^
Peonidin-3-acglc	Anthocyanins	8.43 ± 1.32 ^a^	8.54 ± 1.45 ^a^	8.46 ± 1.61 ^a^
Catechin	Flavanols	7.83 ± 1.17 ^a^	7.69 ± 1.23 ^a^	7.77 ± 1.14 ^a^
Epicatechin	Flavanols	11.42 ± 1.46 ^a^	11.53 ± 1.53 ^a^	11.39 ± 1.77 ^a^
Procyanidin dimer	Flavanols	6.54 ± 0.86 ^a^	6.48 ± 0.98 ^a^	6.41 ± 1.05 ^a^
Myricetin-3-glc	Flavonols	1.35 ± 0.19 ^a^	1.41 ± 0.21 ^a^	1.38 ± 0.17 ^a^
Astilbin	Flavonols	2.41 ± 0.26 ^a^	2.37 ± 0.34 ^a^	2.46 ± 0.23 ^a^
Laricitrin-3-glc	Flavonols	1.79 ± 0.13 ^a^	1.83 ± 0.16 ^a^	1.76 ± 0.17 ^a^
Quercetin-3-glc	Flavonols	2.23 ± 0.22 ^a^	2.19 ± 0.26 ^a^	2.31 ± 0.27 ^a^
Isoquercetin	Flavonols	3.87 ± 0.23 ^a^	3.73 ± 0.27 ^a^	3.84 ± 0.29 ^a^
Syringetin-3-glc	Flavonols	2.88 ± 0.23 ^a^	2.81 ± 0.29 ^a^	2.91 ± 0.21 ^a^
Isorhamnetin-3-glc	Flavonols	4.35 ± 0.34 ^a^	4.39 ± 0.31 ^a^	4.29 ± 0.33 ^a^
Gallic acid	Phenolic Acids	32.8 ± 4.13 ^a^	30.9 ± 3.99 ^a^	31.7 ± 3.82 ^a^
Fertaric acid	Phenolic Acids	27.6 ± 3.89 ^a^	27.9 ± 3.64 ^a^	28.2 ± 4.11 ^a^
Ethyl gallate	Phenolic Acids	18.3 ± 3.02 ^a^	17.9 ± 2.88 ^a^	17.4 ± 2.73 ^a^
Caftaric acid	Phenolic Acids	8.45 ± 1.31 ^a^	8.77 ± 1.28 ^a^	8.93 ± 1.02 ^a^
Coutaric acid	Phenolic Acids	12.46 ± 1.42 ^a^	12.91 ± 1.32 ^a^	12.13 ± 1.19 ^a^

Data show the mean plus standard deviations (*n* = 3). Different letters in the same row show the remarkable differences by ANOVA (*p* < 0.05).

**Table 3:** Antioxidant activity of red wine at different digestion phases.

	DPPH Radical Scavenging Activity (%)		
	Undigested	After Digestion	Transmembrane
Tianzhu	78.4 ± 3.88 ^b^	52.5 ± 2.35 ^b^	23.3 ± 2.21 ^b^
Helanshan	71.5 ± 3.41 ^a^	46.8 ± 2.18 ^a^	20.8 ± 1.98 ^a^
Cabernet	82.7 ± 4.21 ^c^	67.9 ± 2.49 ^c^	15.9 ± 2.43 ^c^
	FRAP radical scavenging activity (%)		
	Undigested	After digestion	Transmembrane
Tianzhu	52.7 ± 2.64 ^b^	49.6 ± 2.51 ^b^	15.3 ± 1.12 ^b^
Helanshan	47.1 ± 2.19 ^a^	41.9 ± 1.78 ^a^	13.4 ± 1.28 ^a^
Cabernet	61.2 ± 2.97 ^c^	55.3 ± 2.85 ^c^	15.6 ± 1.42 ^b^
	ABTS radical scavenging activity (%)		
	Undigested	After digestion	Transmembrane
Tianzhu	72.6 ± 3.66 ^a^	68.1 ± 3.25 ^a^	20.6 ± 1.97 ^a^
Helanshan	72.1 ± 3.85 ^a^	70.6 ± 3.71 ^a^	21.7 ± 2.01 ^a^
Cabernet	95.3 ± 4.78 ^b^	89.3 ± 4.16 ^b^	29.4 ± 2.84 ^b^

Data showed mean plus standard deviations (*n* = 3). Different letters in the same row show the remarkable differences by ANOVA (*p* < 0.05).

## Data Availability

Data is contained within the article.
